# Forkhead Box M1 (FoxM1) Gene Is a New STAT3 Transcriptional Factor Target and Is Essential for Proliferation, Survival and DNA Repair of K562 Cell Line

**DOI:** 10.1371/journal.pone.0048160

**Published:** 2012-10-24

**Authors:** André L. Mencalha, Renata Binato, Gerson M. Ferreira, Barbara Du Rocher, Eliana Abdelhay

**Affiliations:** Laboratório de Células-Tronco, Divisão de laboratórios do CEMO, Instituto Nacional de Câncer, Rio de Janeiro, Brasil; Cincinnati Children's Hospital Medical Center, United States of America

## Abstract

The forkhead box (Fox) M1 gene belongs to a superfamily of evolutionarily conserved transcriptional regulators that are involved in a wide range of biological processes, and its deregulation has been implicated in cancer survival, proliferation and chemotherapy resistance. However, the role of FoxM1, the signaling involved in its activation and its role in leukemia are poorly known. Here, we demonstrate by gene promoter analysis, Electrophoretic mobility shift assay (EMSA) and chromatin immunoprecipitation (ChIP) assays that FoxM1 is a new target of the STAT3 transcriptional activator. Additionally, FoxM1 is transcriptionally dependent on STAT3 signaling activation. Furthermore, we verified that FoxM1 is crucial for K562 cell proliferation, cell cycle checkpoints and viability and could be related to chemotherapeutic resistance. By microarray analysis, we determined the signaling pathways related to FoxM1 expression and its role in DNA repair using K562 cells. Our results revealed new signaling involved in FoxM1 expression and its role in leukemic cells that elucidate cellular mechanisms associated with the development of leukemia and disease progression.

## Introduction

Forkhead box (Fox) genes are a superfamily of evolutionarily conserved transcriptional regulators clustered by the similarities in their Forkhead (FKH) or Winged Helix (WHD) DNA-binding domain. Fox proteins are grouped into sub-classes from FoxA to FoxS. These proteins are involved in a wide range of biological processes, such as development, differentiation, proliferation, apoptosis, migration and invasion [1].

Among the Fox proteins, accumulating evidence has associated FoxM1 overexpression with a wide range of cancers, including breast cancer, colorectal cancer, lung, medulloblastoma, glioblastoma, pancreatic cancer and leukemia [2]–[8]. To support the FoxM1 role in cancer, several groups have examined the cellular effects of FoxM1 overexpression or inhibition. Furthermore, recent data have revealed that FoxM1 is often associated with cancer patients or cell lines that exhibit chemotherapeutic resistance [5], [9]. Therefore, understanding intrinsic FoxM1 regulation and function has become an important target to better comprehend cancer cell proliferation, progression and drug resistance.

Constitutive FoxM1 activation has been shown to play a significant role in cell cycle control. FoxM1 controls the expression of critical genes regulating the G1/S transition, such as SKP1, CCND1 and CSK1, and the G2/M progression, such as CCNB1 and CDC25B [10]. Furthermore, FoxM1 up-regulate AURKA expression, which is essential to mitotic spindle assembly during mitosis [11]. Although some of these data point to a cell cycle regulatory function for FoxM1, recent published data suggest other functions in which it could play a role. However, the understanding of FoxM1 transcriptional activation and the role of FoxM1 as an oncogene is limited.

To date, some studies have revealed that FoxM1 expression can be driven primarily by the Hedgehog signaling pathway in gastric cancer [12], colorectal cancer [13], meningioma [14] and breast cancer [15]. Moreover, FoxM1 has been proposed as a Ras/MEK/MAPK signaling target [16], [17]. Although some data have revealed FoxM1 as regulated by Hedgehog and Ras signaling pathways in solid cancer, FoxM1 regulation in leukemia, mainly in chronic leukemia, is poorly understood.

FoxM1 and STAT3 are often related to cancer and present similar consequences when overexpressed or inhibited [1], [18]. In a recent publication, we demonstrate that STAT3 is crucial to proliferation and inhibits apoptosis in the leukemic K562 cell line [19]. Although the STAT3 protein was first described as a member of the Jak/Stat signaling pathway, in some cancer cells STAT3 is also activated by non-Jak/Stat proteins, such as BCR-ABL, c-Abl, MEK1, Src and Smoothened. This fact links FoxM1 activation to STAT3 signaling [20], [21].

In this study, we sought to characterize the role and relationship between FoxM1 and STAT3 proteins in a cell line with constitutively activated STAT3, known as K562. First, we analyzed STAT3 as a transcriptional factor for FoxM1 gene expression. Additionally, we evaluated the FoxM1 expression profile in a chemoresistant-derived K562/R cell line, which exhibits chemoresistance to imatinib, the most common drug used to treat chronic myeloid leukemia (CML). Finally, to increase our understanding of the role FoxM1 in our cancer model, we analyzed the overall gene expression changes in FoxM1-depleted K562 cells.

## Results

### Identification of STAT3 binding consensus sequences and validation of STAT3 protein binding at the FoxM1 gene promoter

**Figure 1:**
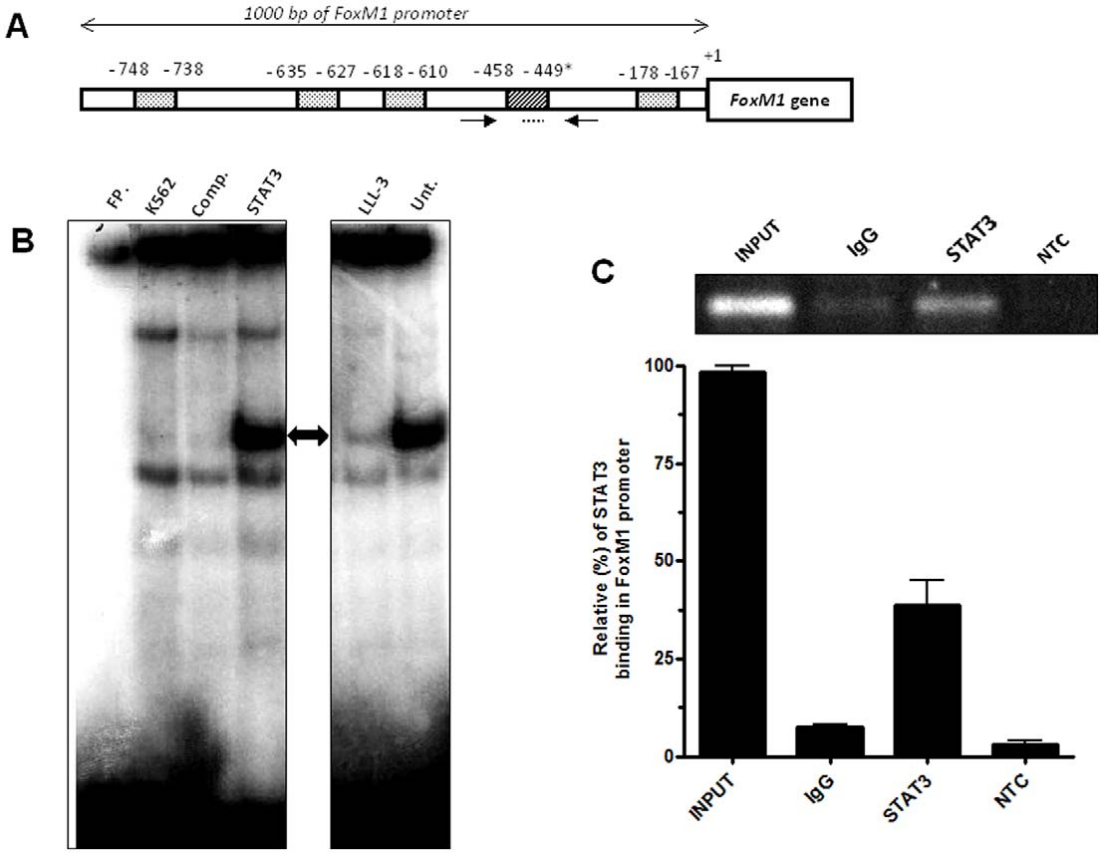
STAT sites and STAT3 interaction with FoxM1 promoter. (A) 1000 bp sequence from FoxM1 promoter gene from start of transcription (+1), indicating STAT bindings sites (doted boxes), STAT3 binding sequence (*, listed boxes). Primer annealing region used to EMSA and ChIP analysis (arrows). (B) EMSA assay, free probe (FP), Nuclear protein extract (K562), Competidor oligo sequence (Comp), STAT3 oligo biding. STAT3 DNA complex (<−>), treatment using LLL-3 (LLL-3), nuclear protein extracts from untreated K562 cells (Unt). (C) ChIP assay, total DNA (Input), IgG and STAT3 immunoprecipiteated DNA (IgG and STAT3), non-template control (NTC).

DNA sequence analysis of 1000 base pairs (bp) from the *FoxM1* promoter revealed five consensus sequences for STAT protein binding (**table** **1**). However, only one of these five putative STAT sites aligns comprehends to the STAT3 binding consensus sequence. The potential STAT3 binding site is located at positions from nucleotide −167 up to −178 bp upstream of the transcription starting site (**figure** **1A**). To verify whether there is STAT3 binding to the STAT3-binding consensus sequences on the *FoxM1* promoter *in vitro* and *in vivo*, electrophoretic mobility shift assays (EMSAs) and chromatin immunoprecipitation (ChIP) assays were performed using a constitutive STAT3 activated cell line, K562. Using the EMSA assay, we validated the STAT3 *in vitro* interaction with a radiolabelled DNA probe designed from the FoxM1 promoter sequence, which contains a STAT3 binding sequence (**figure** **1B**). Furthermore, to confirm the previous results, K562 cells were treated with 40 μM of LLL-3, a STAT3 dimerization inhibitor. STAT3 dimer inhibition abrogated the STAT3-DNA interaction, suggesting specific STAT3 protein binding at the STAT3-consensus sequence from the *FoxM1* promoter (**figure** **1B**). Additionally, the ChIP assay indicated a positive *in vivo* STAT3 interaction with the consensus sequence from the FoxM1 promoter. Using ChIP, we amplified STAT3 in immunoprecipitated DNA fragments and found approximately 35% of the input DNA using primers specific to the FoxM1 promoter DNA sequence (**figure** **1C**). Although the biding sequence of STAT3 is very specific, we evaluated the proximal general STAT biding sites by amplification these regions in the immunoprecipited STAT3 DNA fragments. To these experiments, we amplified a known STAT3 target gene, *CDC25A*, as positive control of immunoprecipitation [22]. Our results shown that our studied STAT3 biding site of *FoxM1*, −440/−432 bp, and of *CDC25A* gene, −222/+58 bp, was preferentially amplified in STAT3 immunoprecipted DNA in comparison to others proximal STAT biding sites (**figure S1**). In summary, all of the experimental assays suggested the binding of the STAT3 protein to the *FoxM1* gene promoter.

**Table 1:** Putative STAT binding sites on FoxM1 gene promoter.

Site number	Location relative to ATG	Consensus sites TTN (4–6)AA
1	−160/−150	TTCCCCCACAA
2	−440/−432	TTAGTCTAA*
3	−600/−593	TTGACTAA
4	−617/−610	TTGCTCAA
5	−730/−721	TTGATTAAAA

* *consensus sequence of STAT3 (TTMN_(4-6)_DAA); M = A or C; D = A, G or T; N = any nucleotide. Transcriptional start site was determined by FoxM1 sequence (NM_202002).*

### FoxM1 gene expression is directly dependent on STAT3 activation

**Figure 2:**
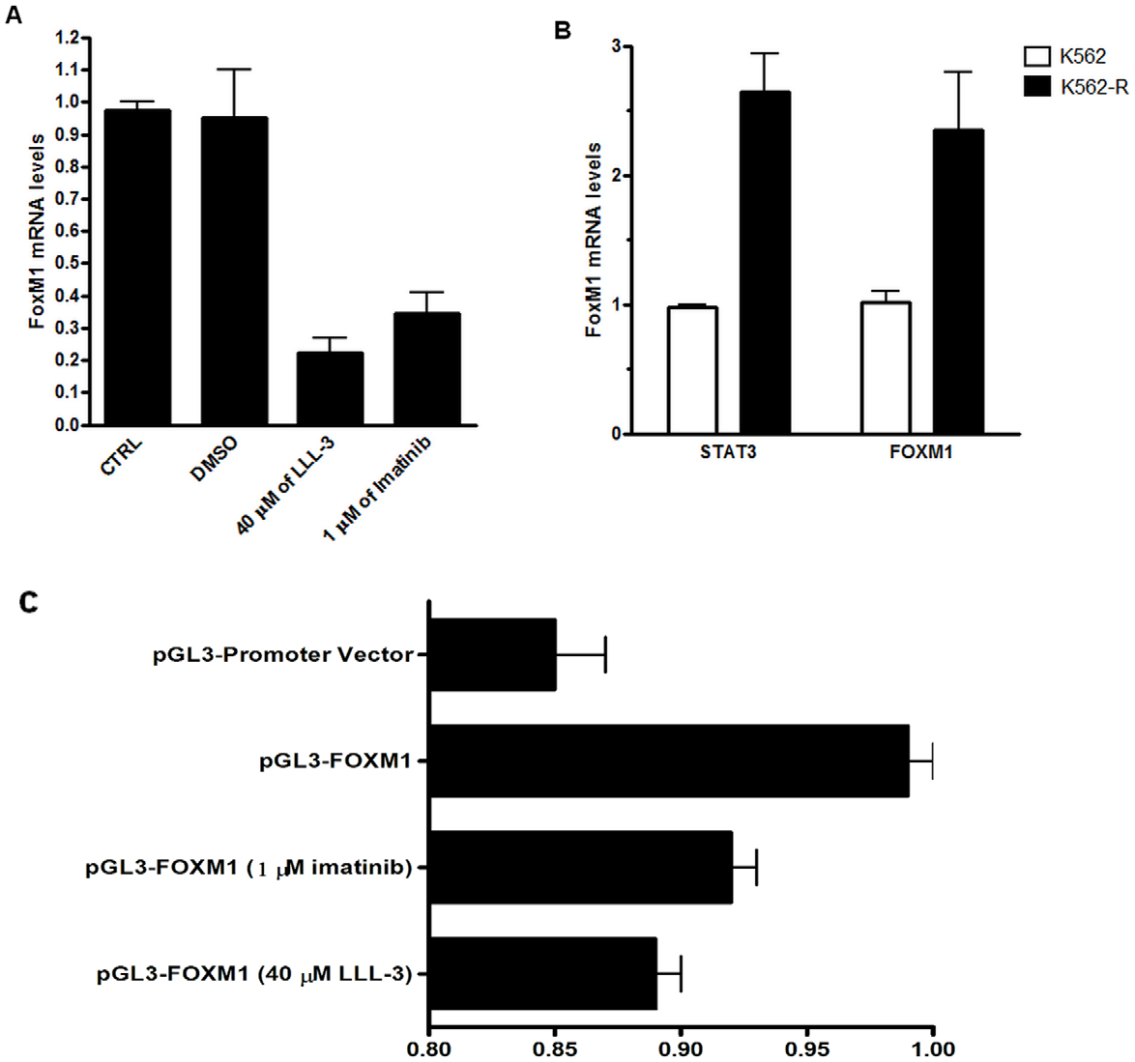
FoxM1 transcription is dependentely of STAT3 and BCR-ABL signaling. (A) Means (± standard deviation) of relative mRNA levels of FoxM1 in response to DMSO, 40 μM of LLL-3 and 1 μM of imatinb treatment after 24 h (Black bars). (B) The relative STAT3 and FoxM1 mRNA levels of K562 (white bars) and K562-R (black bars).

To assess whether STAT3 could serve as a FoxM1 transcriptional activator, we compared the FoxM1 mRNA levels using RT-qPCR following STAT3 inhibitor treatment. The levels of the FoxM1 transcripts were assessed using K562 cells treated with STAT3 inhibitors, LLL-3 to directly inhibit STAT3 or imatinib to indirectly inhibit STAT3 activation by blocking BCR-ABL signaling. At 24 h after treatment with 40 μM of LLL-3 or 1 μM of imatinib, our results indicated that FoxM1 mRNA levels decreased 4-fold in response to the LLL-3 treatment and 3-fold in response to the imatinib treatment when compared to the untreated controls (**figure** **2A**). These results suggest that FoxM1 mRNA levels are dependent on STAT3 activity and BCR-ABL signaling.

To evaluate whether STAT3 is involved in regulating the FoxM1 DNA promoter sequence in vivo, we cloned the FoxM1 DNA promoter region containing the STAT3 binding site, as described in Table 1, into a luciferase reporter gene construct. Our results confirm our hypothesis that STAT3 regulates the transcription of luciferase gene and STAT3 inhibition with LLL-3 or imatinib suppresses the luciferase signal. Cloned FoxM1 DNA promoter increased the luciferase signal by 15% compared to empty vector (**figure** **2C**). Inhibition of STAT3 using 40 µM of LLL-3 or 1 µM of imatinib for 24 h in K562 cells decreased the luciferase signal by approximately 11% and 8%, respectively, compared to untreated cells (**figure** **2C**). Our results indicate that the majority of luciferase signal was promoted by STAT3 transcriptional factor activity, which indicates that STAT3 is an important regulator of the FoxM1 DNA promoter region.

### FoxM1 is crucial to K562 cell proliferation and survival

**Figure 3:**
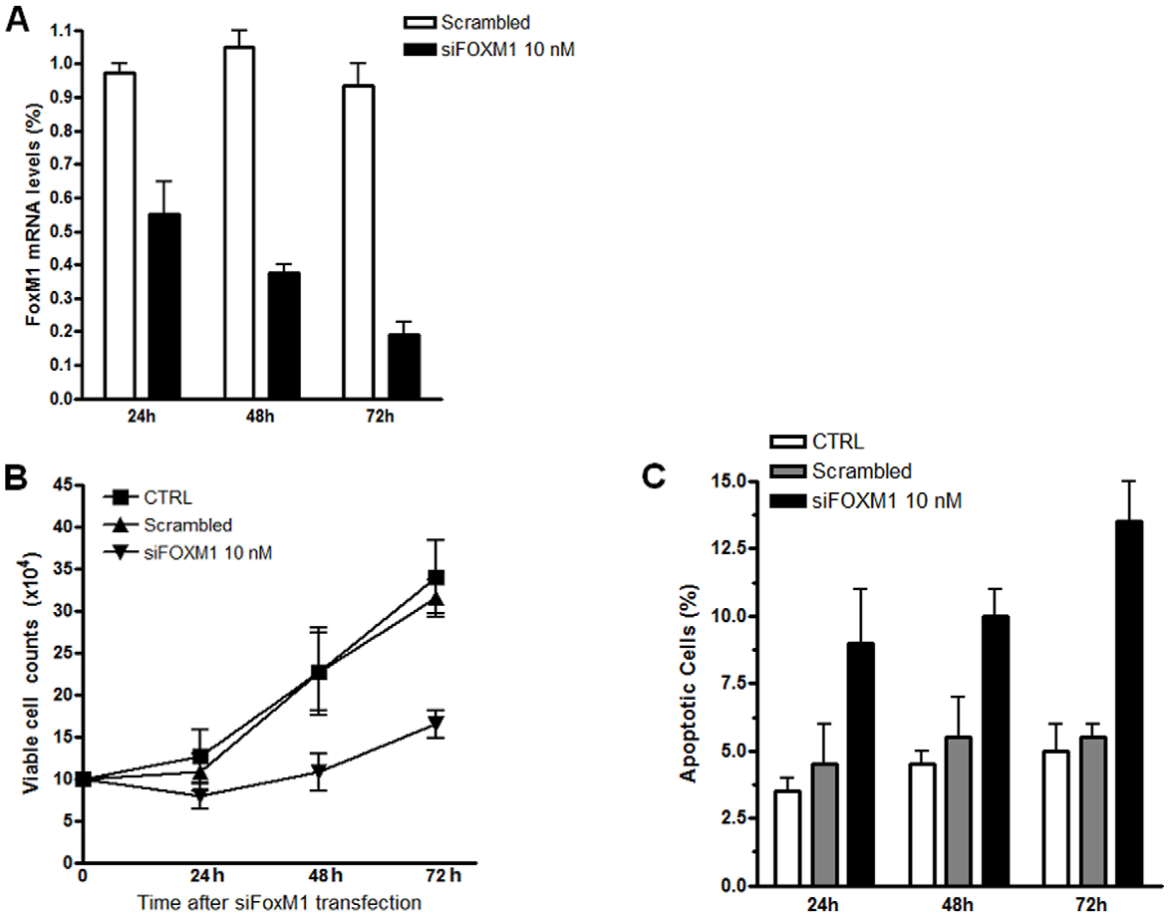
FoxM1 mRNA depletion inhibits proliferation and induces apoptosis. (A) Means (± standard deviation) of relative mRNA levels of inhibition of FoxM1 at 24, 48 and 72 h. (B) Proliferation of K562 cells (CTRL) compared to Scrambled and K562 FoxM1 depleted cells (siFoxM1 10 nM) at 24, 48 and 72 h. (C) Apoptosis analysis of K562 (CTRL, white bars), Scrambled-transfected K562 cells (cian bars) and K562 FoxM1 depleted cells (black bars) at 24, 48 and 72 h.

**Figure 4:**
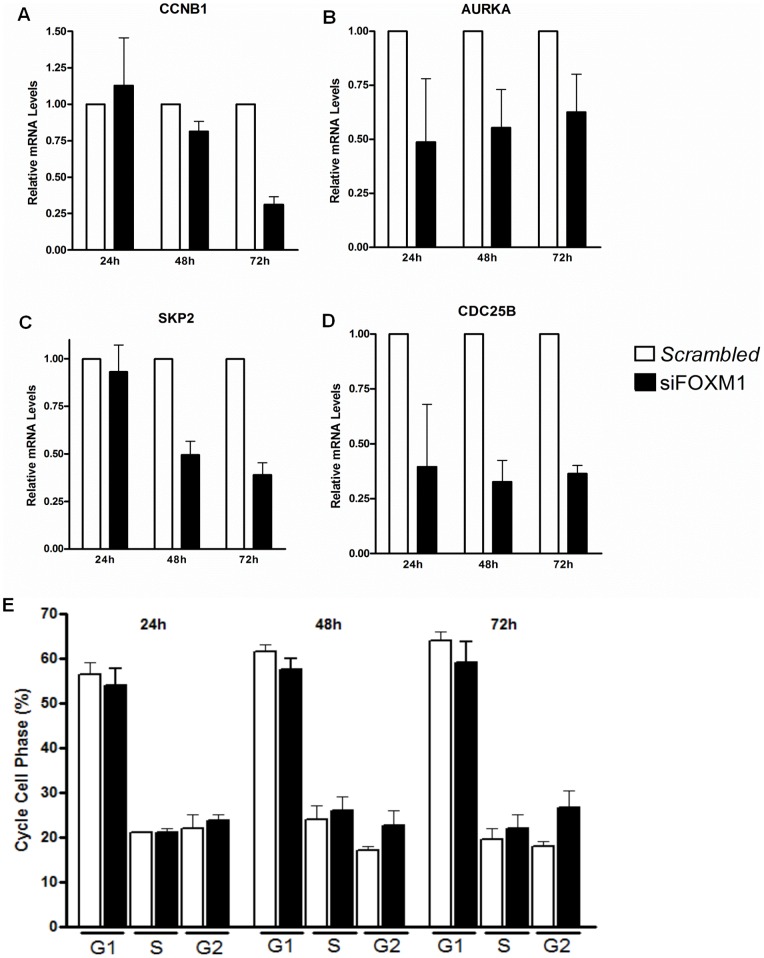
Cell cycle and checkpoint genes are deregulated by FoxM1 inhibition. (A, B, C, D) Means (± standard deviation) of relative CCNB1, AURKA, SKP2, CDC25B mRNA levels of inhibition of K562 FoxM1 depleted cells at 24, 48 and 72 h. (E) Cell cycle content analysis (E), G1 phase, S phase, G2 phase, by Flow Cytometer. Two-way Anova test p<0,05.

To determine whether FoxM1 could be related to the rate of K562 cell proliferation, FoxM1 expression was depleted by a RNA interference (siRNA) assay. First, FoxM1 transcript levels were assessed after transfection by RT-qPCR to evaluate the percentages of inhibition. FoxM1 mRNA levels were significantly inhibited, on average, 60%, 75% and 85% at 24, 48 and 72 h, respectively, when siRNA was used at a 10 nM concentration (**figure** **3A**). We then investigated the effects of FoxM1 on the K562 cell proliferation ratio. Our results showed that FoxM1 inhibition culminates in blocking K562 cell proliferation by approximately 50% and 75% at 48 and 72 h, respectively, compared to the control or scrambled-transfected K562 cells (**figure** **3B**). These results suggest that FoxM1 appears to be crucial for K562 cell proliferation. To determine whether this decrease is related to a loss of viability or cell cycle progression, annexin-V and propidium iodide (PI) tests were performed to assess apoptosis and to evaluate DNA content to determine the cell cycle phases in the FoxM1-depleted K562 cells. In the apoptosis assays, the percentage of apoptotic cells after 24, 48 and 72 hours of silencing was 8% (±2.5%), 10% (±2.15%) and 14% (±3.04%), respectively, when comparing FoxM1 inhibited with scrambled-transfected K562 cells (**figure** **3C**). These data suggest that FoxM1 is essential to K562 cell viability. Our cell cycle analysis showed that the cells remained without significant changes between the cell cycle phases during the transfection periods. Our results indicated that overall, approximately 60% of the cells were in the G1 cell cycle phase, approximately 20% were in the S phase and 20% were in the G2 phase in both the siRNA-scrambled-treated and siRNA-FoxM1-treated cells (**figure** **4E**). At 48 h and 72 h after FoxM1 depletion, we observed a tiny accumulation G2 phase K562 cells compared to siRNA-scrambled-treated. These results suggest that FoxM1 inhibition in K562 cells reduces cell viability and does not promote the accumulation of cells in a specific phase of the cell cycle.

### Chemotherapeutic drug resistant K562 cells exhibit high expression of FoxM1

Elevated expression of FoxM1 has been extensively reported in several solid tumor types and specifically has been closely related to cancer chemotherapeutics resistance [23]. However, the understanding of the role of FoxM1 drug resistance in leukemia is poorly known. To further investigate this issue, we evaluated FoxM1 expression in a chronic myeloid leukemia K562 cell line and in the related resistant cell line, K562-R. We also evaluated the STAT3 mRNA levels to investigate STAT3 expression and its correlation with FoxM1 levels. The relative mRNA levels were measured by RT-qPCR assay. Our tests showed that FoxM1 mRNA was increased 2.7 fold in K562-R cells when compared to K562 cells (**figure** **2B**). Similarly, STAT3 mRNA levels were also elevated by approximately 2.5 fold in imatinib-resistant K562-R cells compared to K562 cells (**figure** **2B**). Similar to existing data concerning solid tumors, our data suggests that FoxM1 and STAT3 mRNA levels are concomitantly overexpressed in leukemia-resistant K562-R cells.

### Microarray analysis reveals the full role of FoxM1 in the modulation of the cell cycle and for transcription of genes involved in DNA repair

**Figure 5:**
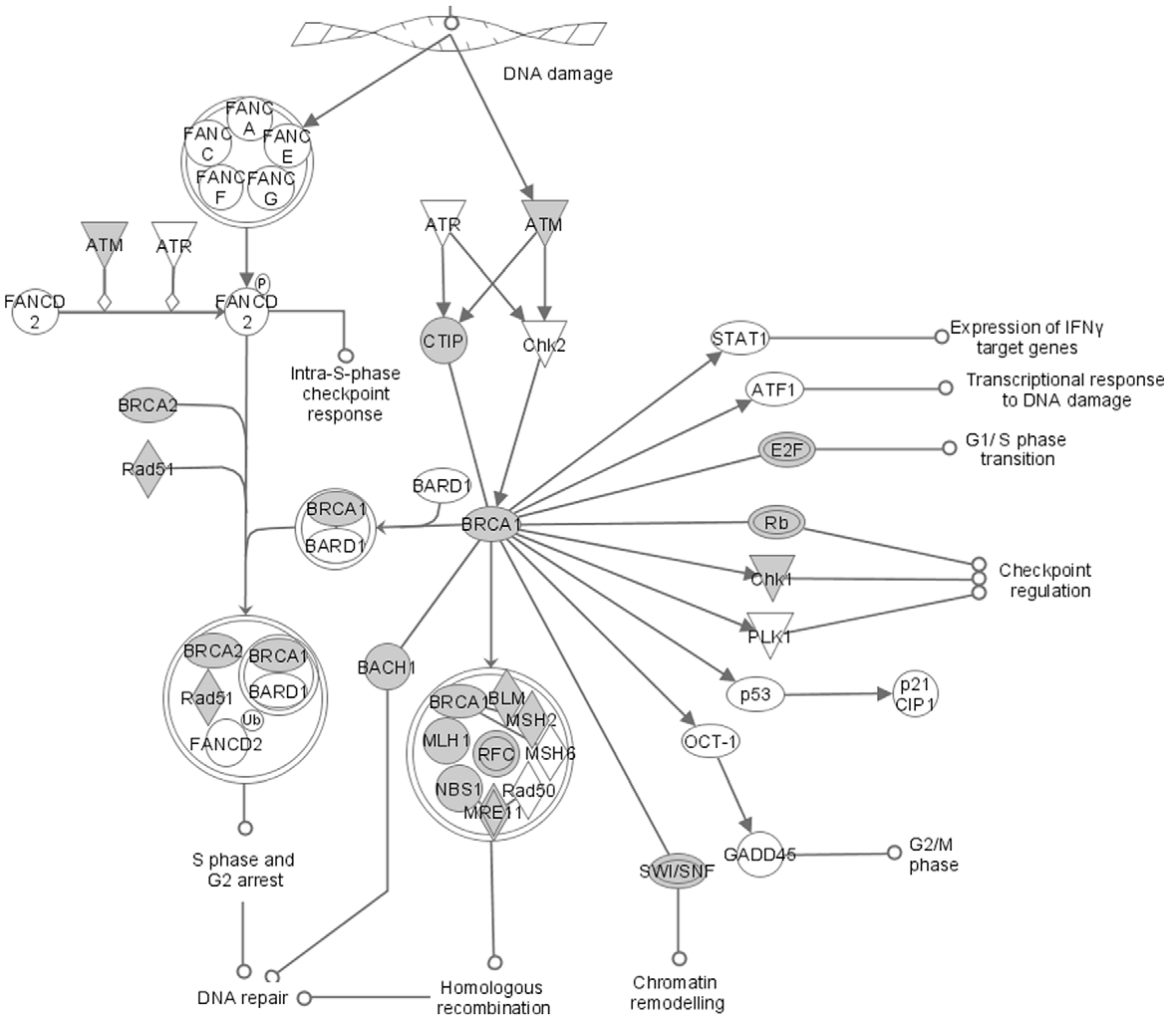
Interacting pathway analysis. Illustration of the most expressed genes in the microarray of FoxM1-depleted cells, which included cell cycle regulators and DNA repair-related pathways. The input list genes are in gray; unfilled forms represent genes that were not part of the input list. The different symbols represent enzymes (diamonds), kinases (triangle), transcription factors (oval), not classified (circle), and protein complex (double circle). The figure was adapted from IPA software (Ingenuity Systems).

To identify the gene targets of the FoxM1 transcriptional factor, we compared the expressed gene changes by comparing the K562 cells depleted of FoxM1 by siRNA with the scrambled-siRNA transfected K562 cells using microarray analysis. The data from the up-regulated and down-regulated differentially expressed genes were submitted to determine the signaling pathway mapping using Ingenuity Pathway analysis (IPA). Our results showed a total of 1668 genes downregulated and 1397 genes up-regulated in K562 cells depleted of FoxM1 when compared to the scrambled-siRNA-transfected K562 cells. We list our chip array results, which includes all genes that are altered as a consequence of FoxM1 interference (**table S1**). Through the IPA analysis, we selected the most significant biological processes and molecular function altered (**tables** **2** **,** **3** **, and figures S3 and S4**). The down-regulated genes were primarily clustered in the cell cycle processes (including cell cycle checkpoints, mitotic assembly and DNA duplication), the DNA repair pathways (including BRCA1 in DNA damage response and ATM signaling), protein ubiquitination and hereditary breast cancer signaling (**Figure S3**). The up-regulated genes were grouped into similar processes, such as cell cycle and DNA repair pathways (**figure** **5** **and figure S4**). However, the up-regulated genes also included genes in the embryonic stem-cell pathways, G protein signaling and others. Our results and analysis indicate that FoxM1 is involved in the regulation of genes from distinct cellular processes, but primarily regulates cell cycle and DNA repair.

**Table 2:** Molecular functions altered by FoxM1 inhibition.

Name	p-value	# Molecules
Cell Cycle	4,95E-11–3,18E-02	232
Cellular Assembly and Organization	1,36E-10–3,18E-02	153
DNA Replication, Recombination, and Repair	1,36E-10–2,97E-02	210
Cellular Function and Maintenance	9,62E-07–3,18E-02	52
Post-Translational Modification	1,25E-06–8,61E-03	175

*Data from IPA software (Ingenuity Systems).*

**Table 3:** Cellular functions altered by FoxM1 inhibition.

Name	p-value	Ratio
Protein Ubiquitination Pathway	6,42E-15	65/274 (0,237)
Role of BRCA1 in DNA Damage Response	1,71E-10	23/61 (0,377)
DNA Double-Strand Break Repair by Homologous Recombination	6,84E-07	9/17 (0,529)
Hereditary Breast Cancer Signaling	7,01E-06	26/129 (0,202)
Mitotic Roles of Polo-Like Kinase	3,46E-05	16/64 (0,25)

*Data from IPA software (Ingenuity Systems).*

### FoxM1 coordinates cell cycle gene expression

To validate the gene expression changes detected by the microarray analysis and to focus on the most changed biological process, the cell cycle, we analyzed the mRNA levels of the genes involved in the cell cycle by RT-qPCR. We evaluated the mRNA level changes of *SKP2* from the G1/S transition, *CCNB1* and *CDC25B* from the G2/M transition and *AURKA* from the mitosis progression. Our results demonstrated that from 24 to 72 hours after the siRNA-FoxM1 transfections, CDC25B and AURKA mRNA levels were decreased by approximately 2 and 3 fold, respectively, compared to the scrambled-siRNA-treated cells (**figure** **4B, 4C**). However, the SKP2 mRNA levels of the siRNA-FoxM1-transfected cells became significantly decreased after 48 to 72 hours by approximately 2 fold in comparison to the scrambled-RNA-treated cells (**figure** **4C**). Moreover, following siRNA-FoxM1 transfection, the CCNB1 mRNA levels were significantly decreased (4 fold) compared to scrambled-RNA-treated cells at 72 hours (**figure** **4A**). The microarray results demonstrate that CCNB1, CDC25B and SKP2 mRNA levels were decreased approximately 2 fold, and the AURKA mRNA levels were decreased approximately 3.2 fold following siRNA-FoxM1 transfection (**figure S2**). Our RT-qPCR results validate the differential changes in mRNA levels found through our microarray analyses because these two methodologies showed similar alterations in the mRNA levels of these genes. Additionally, these data suggest that FoxM1 could act as a transcriptional activator of the genes important to cell-cycle progression.

## Discussion

Several signaling pathways have been involved in CML disease, and STAT3 activation is crucial for the survival and proliferation of CML cells [17], [19]. The majority of CML-active signaling pathways promote direct or indirect STAT3 activation. In this scenario, STAT3 is classically activated by JAK/STAT but can also be activated by the crosstalk of another signal, such as MEK from Ras/Raf/MAPK [20] or by Smoothened from Sonic Hedgehog (Shh) signaling [21].

Additionally, FoxM1 is directly related to cells that have high proliferation rates, such as embryonic and cancer cells, but is mainly expressed in solid tumors [24]. Although FoxM1 has been proposed to be essential for a myriad of solid tumor cancers, the mechanisms that control FoxM1 expression and the role of FoxM1 in leukemia have not been fully elucidated. Because STAT3 and FoxM1 are overexpressed in similar cancer types and coordinate similar cellular mechanisms, we investigated the relationship between FoxM1 and STAT3.

Using *FoxM1* gene promoter analyses, we identified several STAT consensus-binding sequences and one STAT3-specific consensus sequence, which we demonstrated to be functional using EMSA, ChIP and luciferase reporter assay. Moreover, our results determined that FoxM1 expression is STAT3 dependent. To date, FoxM1 has also been reported as a target of the Shh or Ras/Raf/MAPK pathways [17], [23], which are the same pathways that activate STAT3. Therefore, we hypothesize that STAT3 can act as a transducer of Shh and Ras signaling for FoxM1 expression. Therefore, this is the first report that describes *FoxM1* as a direct STAT3 gene target.

The activation of both Shh and Ras pathways, as well STAT3, have been associated with drug resistance in CML [25], [26]. Our K562-R cells, which are more resistant to drug treatment than the K562 cells, exhibited a similar overexpression of both FoxM1 and STAT3. In addition to STAT3, FoxM1 has been associated with drug resistance. Therefore, STAT3 and FoxM1 may be involved in CML drug resistance. However, more investigations are needed to confirm its role in this mechanism. The establishment of FoxM1 as a STAT3 gene target could connect STAT3 signaling to cancer-related cellular processes, such as increased proliferation, survival and drug resistance.

FoxM1 has been primarily related to the transcription activation of cell cycle checkpoints genes, particularly in solid tumors [27], [28]. As observed in solid tumors, we found that FoxM1 is intrinsically related to proliferation and activates cell cycle checkpoints genes in CML cells. Additionally, our data reported a reduction of cell cycle genes by FoxM1 activation. Furthermore, FoxM1-depleted cells did not stop at specific cell cycle phases, although we did observe a slight accumulation of K562 cells in the G2 phase at 48 h and 72 h, which suggests that FoxM1 did not promote dramatic changes in this specific cell line at the observed time point. It is possible that prolonged or stable FoxM1 depletion could affect K562 cell cycle progression. Although we showed that FoxM1 participates in the regulation of G2/M [29], G1/S [30] and mitosis checkpoint gene expression [31], the role of FoxM1 in this cell type may be attenuated by the complex and intricate signaling pathways promoted by BCR-ABL tyrosine-kinase overactivity. FoxM1 has been suggested to be crucial to cell cycle progression in other cancer cells. In acute myeloid leukemia cell lines, FoxM1 is involved in G2/M and S phase checkpoints and enhances proliferation [8]. Therefore, a decrease in FoxM1 expression appear do not interfere in the cell cycle checkpoints of K562 cells but reduce the number of critical genes required for these steps.

In addition to the arrest of cell cycle progression, our data demonstrated a loss of K562 cell viability in the absence of FoxM1. A prolonged cell cycle checkpoint has often been accompanied with a loss of viability by triggering programmed cell death [32]. Although our microarray and pathway analysis results confirmed that FoxM1 primarily regulates the cell cycle, this present study also demonstrate the involvement of FoxM1 in DNA repair in leukemic cells. Recently, FoxM1 signaling was described as essential for coordinating cell cycle progression and DNA repair in ovarian cancer [33]. Furthermore, several data suggest that FoxM1 overexpression promotes genomic instabilities [34], [35]. Our DNA repair pathway was mainly conducted by BRCA1 signaling, which is associated with a non-homologous end-joining repair, considered an error-prone repair [36]. Therefore, FoxM1 overexpression promotes proliferation and the DNA repair mechanisms that allow K562 cell survival and increase genomic instability. Although the role of FoxM1 in cellular proliferation has been extensively described, its function in DNA repair can be expanded to increase the understanding of the role of FoxM1 in maintaining DNA integrity and to understand CML disease progression.

CML is basically subdivided into the chronic phase (CP), the accelerate phase (AP) and the advanced stage, known as the blastic phase (BP); the BP has a poor prognosis and usually is fatal [37]. The progression from CP to BP has been related to genetic instability, which accumulates genetic abnormalities in the course of disease evolution [38]. Although BCR-ABL signaling contributes to CML development, little is known concerning disease evolution. It has been reported that Shh and Smoothened, which are both members of the Shh pathway, were overexpressed in the blastic phase compared to the chronic phase [39]. Because STAT3 and FoxM1 have been directly related to the Shh pathway, we hypothesized that increased genomic instability may be related to increased FoxM1 expression during CML evolution. However, patient analyses must be performed to verify this hypothesis. Therefore, our results indirectly suggest that FoxM1 could be involved in CML disease evolution.

Our study provides the identification of FoxM1 as a new STAT3 gene target and clarifies its role in proliferation, survival, drug resistance and DNA repair in chronic myeloid leukemia. However, the elucidation of the signaling pathways involved in FoxM1 expression in chronic myeloid leukemia might be useful to elucidate new strategies for treatment, drug resistance, prognosis and disease progression.

## Materials and Methods

### Cell lines & Drug treatment

The K562 cell line, established from a CML patient in blast crisis [40], was maintained in RPMI 1640 medium supplemented with 10% fetal bovine serum (Hyclone), 100 U/ml penicillin (Invitrogen), 100 mg/mL streptomycin (Invitrogen) at 37°C in 5% CO2. The K562 cells were used as a BCR-ABL-positive cell line. The establishment of a K562 cell line resistant to the chemotherapeutic imatinib, K562-R, was described by [41]. The drug LLL-3 was used to inhibit STAT3, and imatinib (Novartis) was used to suppress BCR-ABL inhibition. For the treatments, 2×10^5^ cells/mL were exposed to the indicated doses of LLL-3 and imatinib, which were dissolved in dimethyl sulfoxide (DMSO, Sigma Aldrich). The number of viable cells was determined at 24 h and 48 h by trypan blue exclusion. The DMSO-treated cells were used as a vehicle control. LLL-3 was kindly provided by Dr. Pui-Kai Li from Ohio State University, Columbus, USA.

### Real-Time quantification PCR (RT-qPCR)

Total RNA was isolated using TRIzol reagent (Invitrogen), according to the manufacturer's instructions. The purified RNA samples were reverse-transcribed using a SuperScript III First Strand Synthesis kit (Invitrogen) with oligo dT (IDT), following the manufacturer's recommendations. The newly synthesized cDNA was diluted 10-fold in TE (Tris-HCl 10 mM pH 8.0 and EDTA 1 mM) buffer and used for RT-qPCR analysis. The PCR reactions were performed for each sample and analyzed in triplicate. The RT-qPCR was performed using a 1X Power SYBR Green Master Mix, 0.2 μM of each primer and 1.0 μL of the cDNA sample. The reaction was loaded into a Rotor Gene Q (Qiagen), and the following program was executed: an initial cycle at 95°C for 10 min, followed by 45 cycles of 95°C for 15 s and 60°C for 1 min. The relative gene expression levels were determined using the ΔΔCt method (2^−ΔΔCt^) [42]. The *ACTNB* mRNA level was used as a reference to normalize the reactions. The following primers were used: FoxM1 sense: 5′-GACTTCTTGGGTCTTGGGGTG-3′ and antisense: 5′-GGAGGAAATGCCACACTTAGCG-3′; STAT3 sense: 5′-GGGAGAGAGTTACAG.

GTTGGACAT-3′ and antisense: 5′-AGACGCCATTACAAGTGCCA-3′; CCNB1 sense: 5′-GTAATGTTGTAGAGTTGGTGTCC-3′ and antisense: 5′-CATGGTGC.

ACTTTCCTCCTT-3′; AURKA sense: 5′-TCAGTACATGCTCCATCTTCCA-3′ and antisense: 5′-CTCATCATGCATCCGACCTTC-3′; SKP2 sense: 5′-TCCACGGCATACTGTCTCAG-3′ and antisense: 5′-GGGCAAATTCAGAGAATC.

CA-3′; CDC25B sense: 5′-CCTCCGAATCTTCTGATGCAG-3′ and antisense: 5′-GCGTCTGATGGCAAACTGC-3′; ACTNB sense: 5′-TTCCTTCCTGGGCATGGA.

GTC-3′ and antisense: 5′-AGACAGCACTGTGTTGGCGTA-3′.

### Electrophoretic Mobility Shift Assays (EMSA)

For the EMSA experiments, double-strand DNA oligonucleotides were synthesized based on the *FoxM1* sequences from the upstream promoter region, which contained the STAT3 consensus binding site (underlined), FoxM1: 5′-TCAAAGG AACTTAGTCTAATCGGGGGGAGC-3′ (−450/−421 bp from +1 nucleotide). The oligonucleotides were end-labeled with [γ-32P] ATP and T4 polynucleotide kinase (Invitrogen). In the binding reactions, 10 µg of the nuclear protein isolated from a K562 cell line was incubated with 80,000 cpm of a FoxM1 gene promoter sequence, 1 µg of poly (dI:dC) (dI:dC) (GE Healthcare) and 2 µL of binding buffer (50 mM HEPES pH 7.4; 300 mM KCl; 5 mM EDTA and 5 mM DTT, 11.5% Ficoll) in a total volume of 20 µL for 40 min at room temperature (25°C). The untreated K562 protein isolates were used as the control. The reactions were resolved in 4.5% native polyacrylamide gel electrophoresis in 0.5X TBE. In all of the EMSA experiments, the dose chosen for the competitive experiments was in a 200X molar excess. The oligonucleotides for FoxM1 were also used as competitors. For the supershift analysis, 1 µg of anti-STAT3 (Santa Cruz Biotechnologies) antibody was included in the first incubation.

### Chromatin immunoprecipitation assay

To determine the *in vivo* binding of STAT3 to the *FoxM1* promoter DNA sequence, chromatin immunoprecipitation (ChIP) assay was performed [43]. Briefly, 1×10^8^ K562 cells were fixed in 1% formaldehyde for 10 min to crosslink the DNA and the DNA-associated-proteins. The reaction was quenched using 125 nM glycine for 5 min. The cell pellet was washed three times in cold PBS 1X and reconstituted in RIPA buffer containing Protease Mix Inhibitor (Amersham). The cell lysate was sonicated for 3 min with 5 s pulse intervals on a Misonix 3000 sonicator (Misonix), pre-cleared and incubated with 5 µg of anti-STAT3 antibody (sc-482, C20, Santa Cruz) or a normal rabbit IgG antibody (sc-2027, Santa Cruz) followed by an isolation procedure using Protein-A/G Sepharose Beads (GE Healthcare). The beads were washed, and the DNA-protein interaction was reversed by heating to 65°C for 12 h [44]. The DNA was precipitated with ethanol and reconstituted in MilliQ water. The purified immunoprecipitated DNA fragment was amplified by PCR. The primer sequences flanking the STAT3 bind consensus sequence of the *FoxM1* promoter are as follows: 5′-GTAGGGTTCATGGTGCCGACA-3′ and 5′-CGGCTTTAGTTGATTTCCTCAC-3′. The PCR conditions and thermal cycling were performed equally as abovementioned in the “Real-Time PCR” section. The percentage of STAT3 binding (N%) was calculated using the following formula: N%  =  expˆ2^(CtInput – CtSTAT3)^, in which Ct^Input^ and Ct^STAT3^ are the mean PCR cycle thresholds performed in triplicate on DNA samples from the STAT3 and input immunoprecipitations [43]. The PCR products were electrophoresed on 1.5% agarose gel stained with 0.5 µg/mL ethidium bromide for visualization. The following sense primers were used for predicted STAT biding sites: −850 bp 5′-CGGTTTCGCTATGTTGCCAGG-3′; −626 bp 5′-GCACAGCAGTTGCTCAACTAGACT-3′; −466 bp 5′-AGATAATACGCAGCCCTCAAAGG-3′; +1789 5′-GGAGGAAATGCCACACTTAGCG-3′. The following antisense primers were used: −214 bp 5′-GCAGCCGAGGGAGAGTTTG-3′ and +1947 bp 5′-GACTTCTTGGGTCTTGGGGTG-3′. Positive amplification STAT3 biding site for ChIP CDC25A sense: 5′-ATTTTGATCCCCGCTCTTCT-3′ and antisense 5′-GAAAACCAAGCCGACCTACA-3′ [22].

### Luciferase Reporter Assay

Luciferase Reporter Assay: The plasmids used for this experiment are as follows: pGL3-Promoter Vector, pGL3-plasmid containing FOXM1 DNA promoter region (−466/+39) and pRL-TK renilla luciferase expression plasmid was used as an internal control (Promega). The DNA FoxM1 promoter region was amplified using the following primers: FOX1P−466/− sense 5′-AGATAATACGCAGCCCTCAAAGG AAAGG-3′ and FOX1P+39 antisense 5′-TTCTGGCACCGGAGCTTTCAG-3′. PCR products were digested with BglII and KpnI (Promega) and inserted in a pGL3-Promoter Vector using T4 DNA ligase (Invitrogen). The recombinants were transformed and grown. Colonies were confirmed by PCR amplification using pGL3 control primers (Promega), and plasmid minipreparations were performed with a Wizard® Plus SV Minipreps DNA Purification System (Promega). We co-transfected 0.2 µg of pGL3-Promoter Vector or pGL3-FoxM1(−466/+39) with 0.2 µg of pRL-TK Renilla plasmid in K562 cells with Lipofectamine LTX with Plus Reagent (Invitrogen). After 24 h, K562 cells were treated with 40 µM LLL-3 or 1 µM imatinib for 24 h. Luciferase assay quantification was performed with a Dual-Luciferase® Reporter Assay System (Promega) and a Veritas Microplate Luminometer (Promega). Luciferase activity in treated or untreated cells was normalized for transfection efficiency to Renilla activity, and the results were indicated as fold induction in comparison to the cells transfected with empty pGL3-Promoter Vector. All results are representative of at least three independent experiments and represent the mean ± S.D. of triplicate samples.

### RNA interference of FoxM1 transcript

The K562 cells were plated at 1×10^5^ cell/ml in a 24-well plate and left overnight in RPMI-1640 media without antibiotics before the transfections assays. FoxM1 siRNA was synthesized by Integrated DNA Technology (IDT) based on previously published sequences, 5′-GGACCACUUUCCCUACUUU-3′ [8], [45]. A concentration of 10 nM of the scrambled siRNA (SC-37007, Santa Cruz) was used as a siRNA negative control. The transfection efficiency was evaluated using transfections of FITC-conjugated siRNA (SC-36869, Santa Cruz) and analyzed by flow cytometry. The siRNA transfections were performed using 10 nM Trifectin according to the manufacturer's protocol (Promega). The siRNA transfections were conducted for up to 72 hours.

### Cell proliferation, Viability and Cell Cycle Analysis

The proliferation assay was conducted using 1×10^5^ K562 cells/mL of cells transfected with scrambled siRNA or siRNA-FoxM1 in a 24-well plate for 24 h. Subsequently, the cells were stained with 0.1% trypan blue and counted in quadruplicate with a Neubauer chamber. The non-viable cells were excluded by trypan blue staining. The relative number of cells was expressed as a percentage of the siRNA transfections compared with the untransfected cells. Cell viability was evaluated by detecting early apoptosis via annexin V-FITC staining (BD Bioscience) analysis. Briefly, the K562 cells were harvested in 500 µL of binding buffer (10 mM Hepes [pH 7.4]; 150 mM NaCl; 5 mM KCl; 1 mM MgCl_2_ and 1.8 mM CaCl_2_), stained with 1 µL of fluorescein-labeled annexin-V-FITC, followed by a 20-min incubation in the dark. Propidium iodide (1.5 µg/mL) was added to the incubated tubes prior to analysis. Ten thousand events were collected from each sample in a FACSCalibur Flow Cytometer (BD Bioscience). Annexin V-FITC (+), PI (−) cells were considered apoptotic events. Cell cycle analysis was performed by DNA distribution and was analyzed using propidium iodide (PI; Sigma–Aldrich), as described by **Nicoletti et** **al**. The cultured cells were subjected to different treatments and were washed in phosphate-saline buffer, reconstituted in 300 µL of hypotonic buffer (0.1% sodium citrate; 0.1% Triton-X; 100 µg/mL RNase and 50 µg/mL PI) and incubated for 30 min at 4°C.

### Microarray assay and results analysis

Total RNA was extracted using TRIzol Reagent (Invitrogen) and purified by RNeasy (Qiagen) according to the manufacturer's instructions. The total RNA (1 μg) was reverse transcribed and then used to produce biotinylated cRNA using a GeneChip whole transcription (WT) sense target-labeling assay (Affymetrix). The biotinylated cRNA was then hybridized to the GeneChip human exon 1.0 ST array (Affymetrix), which was washed and stained according to the manufacturer's protocols. The GeneChip arrays were scanned using a GeneChip® Scanner 3000, and Affymetrix Expression Console Software Version 1.0 was used to create the summarized expression values. The data were analyzed using Partek® software (http://www.partek.com); a differential expression of at least a 2-fold change was used to define up- and down-expression. Functional grouping and pathway analysis from differential expressed genes were processed and visualized by Ingenuity Pathways Analysis (IPA) (Ingenuity® Systems).

## Supporting Information

Figure S1 **PCR amplification of predicted STAT3 binding sites from immunoprecipted FoxM1 DNA promoter.** Bars represents the means (± standard deviation) of FoxM1 DNA promoter amplification regions from −850/−214 base pairs (bp – green bar); −626/−214 bp (pink bar); −466/−214 bp (purple bar). Positive control of STAT3 ChIP, promoter of CDC25A gene (−222/+48 bp – dark blue bar); Negative control of ChIP, internal DNA sequence of FoxM1 gene (+1789/+1947 bp – light blue bar). IgG: immunoglobulin G.
(TIF)

Figure S2 **Comparison and confirmation of altered genes found in RT-qPCR and Microarray from siRNA od FoxM1 gene:** Bars represents the means (± standard deviation) of relative mRNA levels of CCNB1, CDC25B, SKP2 and AURKA genes. Scrambled siRNA (white bars); RT-qPCR assay (black bars); Microarray assay (gray bars).
(TIF)

Figure S3 **Biofunctions downregulated by FoxM1 inhibition.** Bars represents the most representative biological functions regulated negatively in response to depletion of FoxM1 by siRNA. Data was adapted from from IPA software (Ingenuity Systems).
(TIF)

Figure S4 **Biofunctions upregulated by FoxM1 inhibition.** Bars represents the most representative biological functions regulated positively in response to depletion of FoxM1 by siRNA. Data was adapted from from IPA software (Ingenuity Systems).
(TIF)

Table S1 **Excel file with list of altered genes in FoxM1 siRNA assay.**
(XLS)
