# Supplementary figures and images for: Forkhead Box M1 (FoxM1) Gene Is a New STAT3 Transcriptional Factor Target and Is Essential for Proliferation, Survival and DNA Repair of K562 Cell Line
Source: PLoS One. 2012 Oct 24;7(10):e48160. doi: 10.1371/journal.pone.0048160 (PMC3480485; doi:10.1371/journal.pone.0048160)

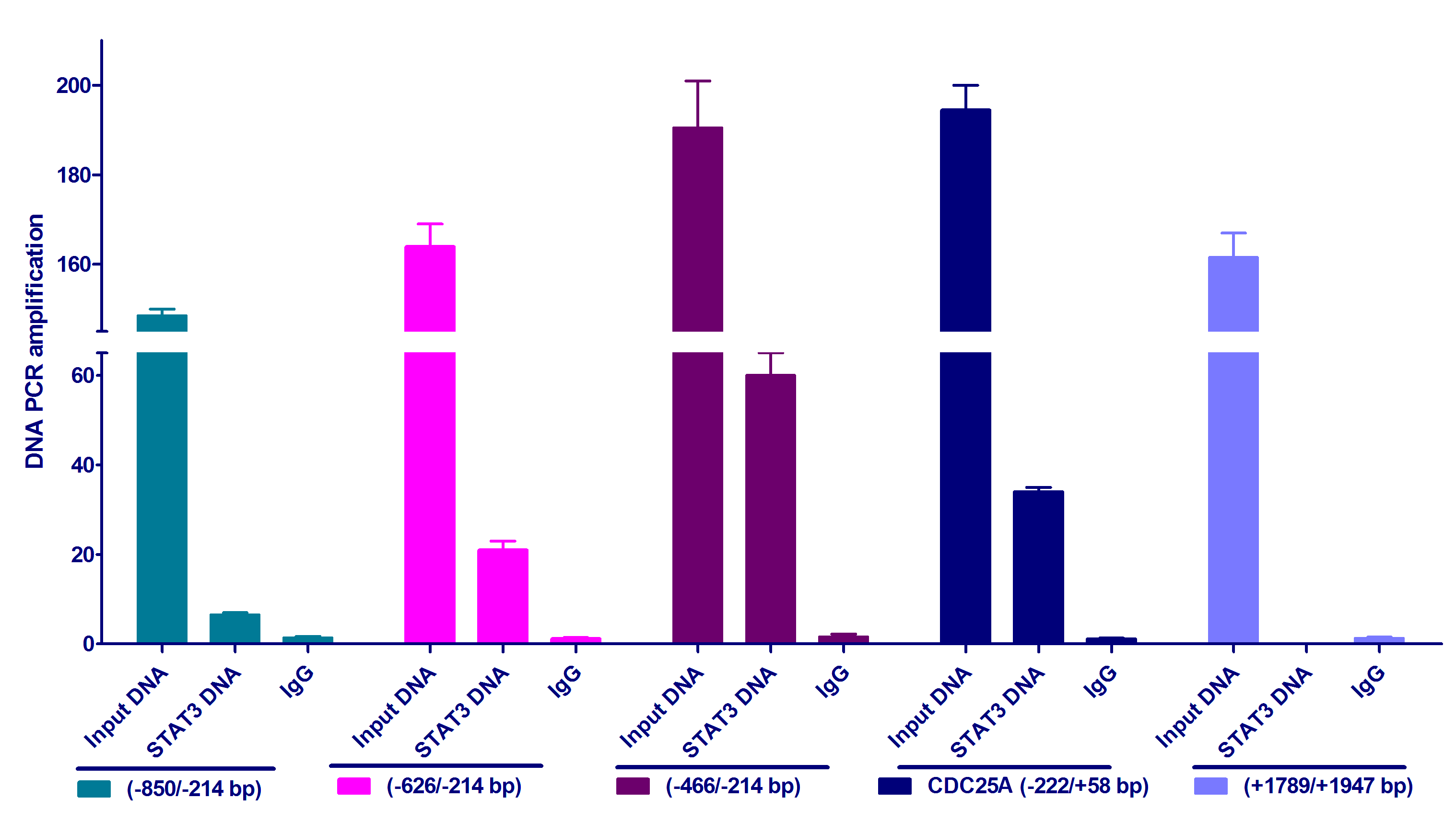

Supplement: Figure S1 — PCR amplification of predicted STAT3 binding sites from immunoprecipted FoxM1 DNA promoter. Bars represents the means (± standard deviation) of FoxM1 DNA promoter amplification regions from −850/−214 base pairs (bp – green bar); −626/−214 bp (pink bar); −466/−214 bp (purple bar). Positive control of STAT3 ChIP, promoter of CDC25A gene (−222/+48 bp – dark blue bar); Negative control of ChIP, internal DNA sequence of FoxM1 gene (+1789/+1947 bp – light blue bar). IgG: immunoglobulin G. (TIF) [file pone.0048160.s001.tif]

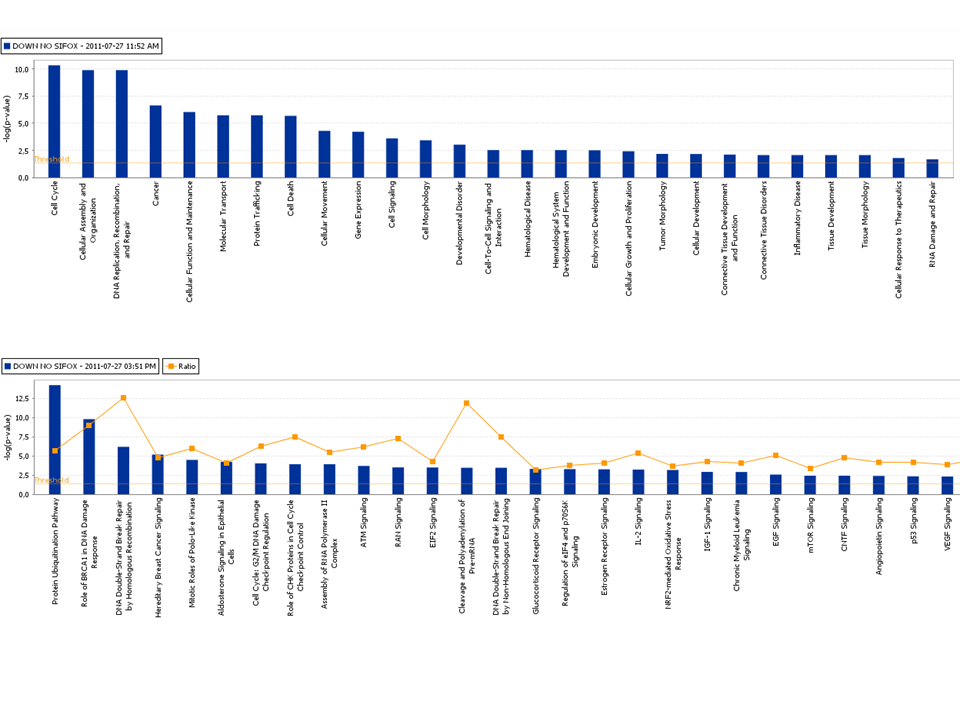

Supplement: Figure S3 — Biofunctions downregulated by FoxM1 inhibition. Bars represents the most representative biological functions regulated negatively in response to depletion of FoxM1 by siRNA. Data was adapted from from IPA software (Ingenuity Systems). (TIF) [file pone.0048160.s003.tif]

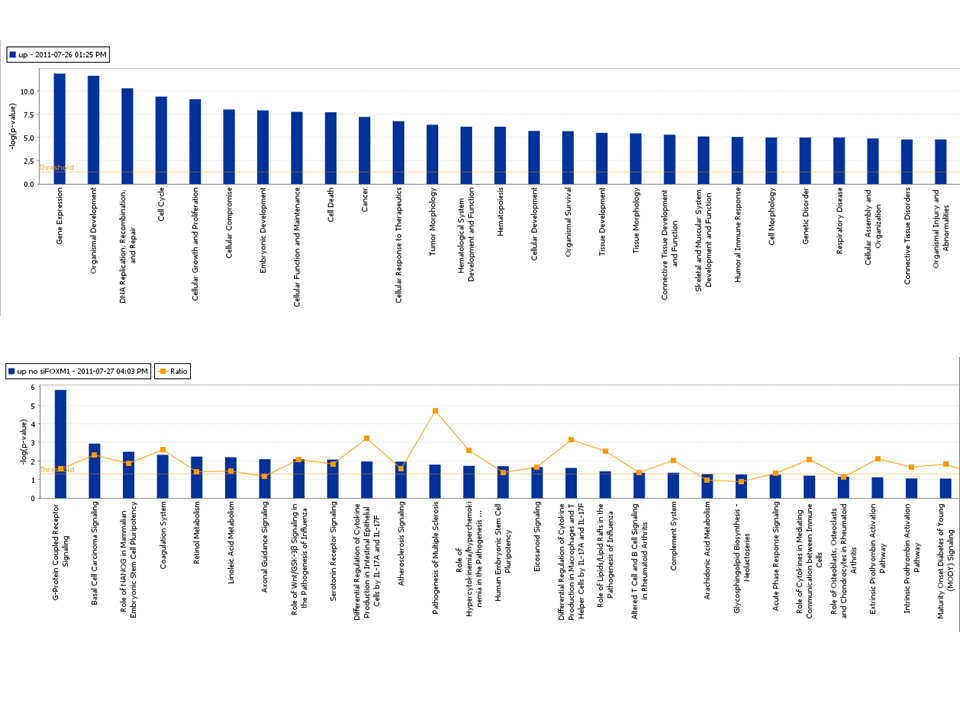

Supplement: Figure S4 — Biofunctions upregulated by FoxM1 inhibition. Bars represents the most representative biological functions regulated positively in response to depletion of FoxM1 by siRNA. Data was adapted from from IPA software (Ingenuity Systems). (TIF) [file pone.0048160.s004.tif]
